# Trait Empathy Modulates Patterns of Personal and Social Emotions During the COVID-19 Pandemic

**DOI:** 10.3389/fpsyg.2022.893328

**Published:** 2022-06-10

**Authors:** Yaji He, Jiajia Zhu, Xuhai Chen, Yan Mu

**Affiliations:** ^1^CAS Key Laboratory of Behavioral Science, Institute of Psychology, Chinese Academy of Sciences, Beijing, China; ^2^Department of Psychology, University of Chinese Academy of Sciences, Beijing, China; ^3^Key Laboratory of Behavior and Cognitive Psychology in Shaanxi Province, School of Psychology, Shaanxi Normal University, Xi’an, China

**Keywords:** COVID-19, threat, emotion, empathy, representational similarity analysis

## Abstract

The COVID-19 pandemic has caused profound consequences on people’s personal and social feelings worldwide. However, little is known about whether individual differences in empathy, a prosocial trait, may affect the emotional feelings under such threat. To address this, we measured 345 Chinese participants’ personal emotions (e.g., active, nervous), social emotions (i.e., fearful and empathetic feelings about various social groups), and their empathy traits during the COVID-19 pandemic. Using the representational similarity analysis (RSA), we calculated the pattern similarity of personal emotions and found the similarity between the positive and negative emotions was less in the high vs. low empathy groups. In addition, people with high (vs. low) empathy traits were more likely to have fearful and sympathetic feelings about the disease-related people (i.e., depression patients, suspected COVID-19 patients, COVID-19 patients, flu patients, SARS patients, AIDS patients, schizophrenic patients) and showed more pattern dissimilarity in the two social feelings toward the disease-related people. These findings suggest a prominent role of trait empathy in modulating emotions across different domains, strengthening the polarization of personal emotions as well as enlarging social feelings toward a set of stigmatized groups when facing a pandemic threat.

## Introduction

COVID-19 as a public health crisis has posed a threat to public mental health and social harmony. It has been changing us greatly by adding uncertainty and loss of control to our lives, each of which is known to trigger emotional dysregulation and distress, such as depression anxiety (Margetić et al., 2021; Tyra et al., 2021). It is no doubt that the public has been suffering from the personal domain of emotional burdens when facing such a threat. Differentiating from personal emotions which represent individuals’ subjective feelings of their own emotional experiences (Thompson, 1991; Gilbert, 2014; Liu and Chen, 2021), the public’s social emotions——the feelings about other people and social groups——are influenced by the social isolation during the COVID-19 pandemic. For instance, accumulating research has revealed that people are afraid of and try to avoid a wide range of social groups, particularly the COVID-19 related people and groups, including healthcare workers, COVID-19 patients, people who recovered from COVID-19, and residents living in high-risk regions (Bagcchi, 2020; Roberto et al., 2020; Abuhammad et al., 2021). Though pandemic has negative impacts on both personal and social emotions, so far, little is known about whether individuals may differ in the two domains of emotions in the context of threat.

It has been well-documented that individual and group differences in cognitive control, emotion-related traits, and biological sex can modulate emotions and the underlying supportive substrates (Tamir et al., 2020). Among these factors, the prominent role of empathy in emotion perception (Olderbak and Wilhelm, 2017), emotional regulation [e.g., cognitive reappraisal, and rumination, Knight et al. (2019) and Zaki (2020)], and sharing others’ emotional feelings [e.g., sad and happy, Spencer-Rodgers et al. (2010)] has been widely discussed. So far, little is known about whether such individual trait may affect personal and social emotions under the enormous ecological threat. The current study is set out to test whether and how trait empathy would affect individuals’ personal and social emotions during the COVID-19 pandemic.

### Empathy and Personal Emotions

Empathy—the capacity to share and understand others’ emotional states—is highly related to the subjective experience of emotions and mirroring others’ emotions (Decety and Jackson, 2006). Prior studies found that people with high relative to low empathy could capture negative emotions more quickly and experience them more deeply (Yan et al., 2016; Sun et al., 2018). Using facial electromyography, researchers further revealed that high (vs. low) empathy individuals induced increased corrugator supercilii activity in processing disgusting and fearful facial expressions, suggesting increased sensitivity of negative emotions in high empathy individuals (Rymarczyk et al., 2016). These findings suggest that high relative to low empathy people show more emotional reactions (e.g., more negative emotions and physiological responses) to process emotional stimuli conveying threatening/negative information (e.g., fear). However, little attention has been paid to the role of empathy in the modulation of emotions under a pandemic threat. Recently, a few studies uncovered that when compared to low empathy people, high empathy people experienced more emotional disorders during the COVID-19 pandemic [e.g., anxiety, depression, and poor sleep quality, Guadagni et al. (2020) and Petrocchi et al. (2021)]. Similarly, the association between empathy and negative emotions (e.g., anger) was replicated in an Eastern sample of 453 Chinese during COVID-19 (Ma and Wang, 2021). So far, most of these studies have focused on the impact of empathy on negative emotions, e.g., vigor, depression, distress (Guadagni et al., 2020; Van de Groep et al., 2020; Grignoli et al., 2021). Considering that positive emotions are of great significance in fostering subjective well-being (Livingstone and Srivastava, 2012) and help promote psychological resilience when facing an ecological threat [e.g., Yamaguchi et al. (2020) and Gurvich et al. (2021)], we are curious about whether empathy may help boost the public’s positive emotions to relieve stress. Furthermore, we are interested in whether empathy may modulate the positive and negative dimensions of personal emotions, the characteristics of the two dimensions (i.e., pattern similarity), and the relationship between the two dimensions.

The relationship (i.e., the ratio) of the two dimensions of personal emotions has been found to function as an indicator of psychological well-being (Larsen, 2009). Toward a better understanding of the relationship between positive and negative emotions, researchers have proposed different theoretical models and debated for a long time [e.g., the bipolar vs. bivariate models, Larsen et al. (2001); Schimmack (2001), and Ong et al. (2017)]. The bipolar model holds the view that positive and negative emotions are opposite to each other, that is, positive emotions and negative emotions are mutually exclusive and opposite to each other, and an individual can experience only one of them at the same time (Russell and Carroll, 1999; Liu et al., 2008). On the contrary, the bivariate model suggests that the two are not mutually exclusive (Larsen et al., 2001), and they could coexist and negatively correlate with each other (Liu et al., 2008). One possible account behind the debate could be that the sources (e.g., personal affairs and social contexts) that trigger individuals’ emotions are complex and diverse rather than singular. Considering that the complexity of determining the sources creates obstacles to understanding emotions and their patterns, it is worth mentioning that COVID-19, as a pandemic threat that has heightened the public’s collective emotions (Stanley et al., 2021), could provide a shared social context and contributes to a group perspective for understanding the relationship between positive and negative emotions and its underlying characteristics (i.e., pattern).

A previous study has shown that when exposed to a stressful event (e.g., a stressful speech), individuals’ positive and negative emotions exhibited more polarization—a higher level of negative correlation (Zautra et al., 2000). This finding suggests that a stressful event could lead to attentional narrowing, which may in turn influence personal emotional states to jointly help humans respond rapidly when facing threats (Hermans et al., 2014). Considering that COVID-19 is a severe stressor that has posed psychological burdens (Elbay et al., 2020; Gritsenko et al., 2020), we speculated COVID-19 might lead to a negative association between positive and negative emotions. Given that individuals with high (vs. low) empathy are more sensitive to negative social events (Yan et al., 2016; Sun et al., 2018), we predicted that people with high (vs. low) empathy might be more likely to show more polarization in the relationship between the two personal emotions.

### Empathy and Social Emotion

Although previous studies have shown that people felt fearful about people and groups related to the COVID-19 disease, such as fear of interacting with people suffering or recovered from COVID-19, toward people related to COVID-19 (Bagcchi, 2020; Roberto et al., 2020; Abuhammad et al., 2021). Meanwhile, some studies have revealed that people would also have positive (or prosocial) feelings, i.e., sympathy, about people related to COVID-19 (Li et al., 2020). The emotional feelings toward others mentioned above are real (or expected) emotional experiences and reactions that people generate in real (or imagined) interactions with others, which are referred to as social emotions (Mercer, 2014). According to the previous research, we speculate that in the face of the pandemic, individuals’ social emotions toward disease-related groups are complex; that is, negative social emotions (fear) and positive social emotions (sympathy) coexist.

Previous evidence has shown that the psychological process of people’s social feelings toward others is closely related to their ability to empathize (Schipper and Petermann, 2013). Based on the definition of the two components of empathy, cognitive empathy reflects an individual’s ability to understand others’ emotions, and affective empathy refers to an individual’s capacity to share others’ emotions (Deutsch and Madle, 1975; Cox et al., 2012). High empathy people therefore may be better at sharing others’ emotions and understanding others’ situations. For example, high compared to low empathy individuals were more willing to offer money and time to assist people with difficulties (Lay et al., 2020; Xiao et al., 2021). Moreover, empathy-related neural activity (i.e., medial prefrontal cortex) contributed to the subsequent empathic concern toward the victim in need (Masten et al., 2011; Spencer-Rodgers et al., 2010; Perez-Bret et al., 2016). Consistently, high empathy people tend to predict high prosocial behavior under the COVID-19 threat (Taylor et al., 2020; Lv et al., 2021; Seong-Wook et al., 2021). Accordingly, we speculated that individuals with high vs. low empathy might generate more prosocial emotions (i.e., sympathetic) toward people in need (e.g., COVID-19 patients) during COVID-19.

On the other hand, recent evidence suggests that empathy may account for people’s fear and avoidance of disease-related groups who may carry the virus during the COVID-19 pandemic. For example, people tried to identify people who might pose a potential infection risk when facing a pandemic threat (Troisi, 2020). In line with this, high empathy people were observed to show more self-protection tendencies during COVID-19, such as engaging in physical distancing and wearing facial masks (Sassenrath et al., 2021). It is assumed that more fear of the disease-related groups and increased self-protection focus among high relative to low empathy people are served as a protective mechanism, which supports keeping away from danger when facing the threat of COVID-19 (Ramaci et al., 2020). Though the two seemingly contradictory aspects (i.e., fear and sympathy) of social emotions are related to empathy, so far, it remains unclear whether or to what extent empathy may simultaneously affect the two social emotions toward disease-related groups and their relationship under a pandemic threat.

### The Current Study

The current study aims to examine the impact of empathy on personal and social emotions and their patterns under the threat of COVID-19. Previous research mostly focused on the mean or sum value of different items within the two-valence domains [i.e., calculate two values to represent positive and negative emotions, e.g., Gan and Fu (2022) and Matiz et al. (2022)]. Thus, it may hardly capture whether individuals’ emotions, as well as their feelings about others, may differ in their pattern characteristics in terms of similarity (Riberto et al., 2019). The above issues can be easily addressed by using representational similarity analysis (RSA)—a computational technique that utilizes pairwise comparisons of units to reveal the similarity pattern among a set of variables (Haxby et al., 2014). Unlike the traditional linear correlation testing of two-dimensional values, RSA has been gradually used in understanding behavioral as well as neural patterns in the field of social psychology (Brooks and Freeman, 2018), which helps uncover the pattern similarity and further provides direct comparisons across conditions (Luo et al., 2021).

In the current study, taking advantage of RSA, our first goal was to examine the pattern similarity of the public’s personal emotions (i.e., positive and negative emotions) and the pattern similarity between the two types of emotions during the COVID-19 pandemic. To address this question, we measured Chinese positive and negative emotions (e.g., inspired, active, nervous, and scared) when they were facing the COVID-19 pandemic. Our second goal was to reveal the pattern similarity of the public’s social feelings toward a variety of social groups (e.g., disease-related people, people violating moral norms, and healthy people) during the COVID-19 pandemic. As for social feelings, we first focused on the two sympathetic and fearful feelings toward the COVID-19-related people and groups (e.g., people infected by COVID-19, people who were suspected patients of COVID-19, and people who have been recovered from COVID-19), which have been demonstrated to be stigmatized during this pandemic [e.g., Baldassarre et al. (2020) and Ransing et al. (2020)]. In addition, we were curious about whether their feelings toward the other disease-related groups, such as people suffering from an infectious disease (e.g., AIDS) and people with mental illness (e.g., schizophrenic patients) may show more pattern similarity. Researchers believe that disease-related groups are usually viewed as social deviants who violate certain norms by the public (Phelan et al., 2008). We, therefore, included people who violate the moral norms (e.g., robbers). Finally, we added non-disease people (e.g., healthy people, natives) as control. Last but not the least, the third goal of the study was to test whether people who differ in the prosocial propensity (i.e., trait empathy) may elicit dissimilar patterns of personal and social emotions to help policymakers understand collective emotions in the context of such a crisis. To address this, we measured participants’ trait empathy using a Chinese version of the Interpersonal Reactivity Index (IRI) Scale (Zhang et al., 2010) and compared the pattern similarity of personal and social emotions between the high and low empathy people.

## Materials and Methods

### Participants

We collected two waves of data during the recovery period of COVID-19 (one is from May 2 to July 12, 2020; and the other is in December 2021, the severity of the outbreak is similar to the former in terms of the number of locally confirmed cases in China), using convenience sampling method (via WeChat spreading) through the online platform like Qualtrics.^1^ According to Meade and Craig (2012)’s recommendation for detecting careless data, we ruled out data with wrong answers in the probe questions (e.g., if you notice this question, please select the option “6”; if you choose any other options, your data will be invalid) (*N* = 32) and invalid data (only completed the informed consent part of the questionnaire) (*N* = 21). We excluded 21 participants from Hubei Province, considering that their perceptions of COVID-19 related groups might be different from those who were from other Chinese provinces because the Hubei province had the most serious outbreak of COVID-19 in China during the period that we conducted this study, and people in Hubei had mostly experienced lockdown. Additionally, previous research has shown that people in the areas with severe epidemics have different risk perceptions of the COVID-19 pandemic than those from other areas (Wen et al., 2020). The final sample was 345 (120 males and 224 females, mean age 25.13 ± 7.85 years). Before the survey, all participants were given informed written consent. The present research was approved by the ethics committees of the Institute of Psychology, Chinese Academy of Sciences the institute. The current research was from a big project on COVID-19 and mental health. The dataset of social emotion was from a previously published study (Zhu et al., 2022). In the current study, we reanalyzed the data using representation similarity analysis to address the empathy modulation on emotions, which was not investigated by the previous study.

### Measurements

#### Personal Emotion

Using an adapted Watson et al. (1988)’s Positive and Negative Affectivity Schedule, we measured participants’ positive and negative emotions within a week during the pandemic on a 7-point Likert scale (1 = *never*, 7 = *always*), which included four positive (i.e., interested, proud, inspired, active) and seven negative emotions (i.e., upset, irritable, nervous, hostile, jittery, guilt, scared). The selected items have been proven to be related to individual emotional states under the pandemic threat [e.g., upset, and sacred in Hennein and Lowe (2020); nervous in Wang et al. (2020)]. The Cronbach’s α coefficients were 0.81 for the positive and 0.90 for the negative dimension.

#### Social Emotion

We measured subjective reports on the possibility of participants’ positive (sympathetic) and negative (fearful) feelings toward a variety of social groups on a 7-point scale (1 = *not at all*, 7 = *extremely*). There were 15 social groups, including healthy people, natives, outsiders, Muslims, depression patients, recovered COVID-19 people, suspected COVID-19 patients, COVID-19 patients, flu patients, SARS patients, AIDS patients, schizophrenic patients, people with masks, people not wearing masks, and robbers. The above social groups have been extensively studied in previous studies on social feelings and attitudes [e.g., Thornicroft et al. (2009); Nyblade et al. (2018), Javed et al. (2021), and Reinius et al. (2021)]. Hierarchical cluster analysis was adopted for grouping the 15 groups into three clusters: the disease, the control, and the social deviant (Supplementary Figure 1). In the hierarchical clustering analysis, we used the *hclust* function in the corrrplot package of R (Wei et al., 2017), which can directly perform hierarchical clustering and visualization of the results. Based on the cluster analysis, the following targets, including depression patients, suspected COVID-19 patients, COVID-19 patients, flu patients, SARS patients, AIDS patients, and schizophrenic patients, were incorporated into one unit as the disease cluster (Supplementary Figure 1). Similarly, these groups (i.e., healthy people, natives, outsiders, people with masks) were clustered into one unit as the non-disease control cluster (Supplementary Figure 1), and the remaining groups were labeled as the social deviant cluster. Considering the variances in the deviant cluster and our research scope, we mainly focused on the differences between the disease and the control clusters.

#### Trait Empathy

We used a Chinese version of the Interpersonal Reactivity Index (IRI) Scale (Zhang et al., 2010) with nine items to measure participants’ ability to share and understand others’ feelings on a 7-point scale (1 = *fully disagree*, 7 = *fully agree*) (e.g., “I try to look at everybody’s side of a disagreement before I make a decision”). This scale included the four dimensions as the original IRI scale (Davis, 1980), including two cognitive empathy (empathetic concern, perspective-taking) and two affective empathy (personal distress, and fantasy) dimensions. We calculated the correlation of affective empathy (α = 0.71) and cognitive empathy (α = 0.76) and the two were correlated (*r* = 0.49, *p* < 0.001). Therefore, we merged the four dimensions and calculated the mean score of the IRI scale for each participant. A higher score indicated a higher level of trait empathy. We then split participants into two groups: high (above-average, *N* = 171) and low empathy (below-average, *N* = 174) groups. The Cronbach’s α coefficient was 0.74.

### Data Analysis

#### Distribution of Personal and Social Emotions

To examine the distribution of personal and social emotions, we first applied the Kolmogorov-Smirnov test. Next, we ran the Mann-Whitney *U*-test to examine the differences in the distribution of the two types of emotions between the high and low empathy groups.

#### Representation Similarity Analysis

According to previous research (Luo et al., 2021), we first calculated the representation similarity matrix (RSM) on the personal and social emotions of all participants. Specifically, we calculated the representation similarity matrix (RSM) on the seven negative emotions (negative-emotion RSM) across all participants using the Pearson correlation coefficients. The same method was used for generating the RSM of positive emotions (positive-emotion RSM). Furthermore, to investigate the relationship between the positive and negative emotions, we calculated the RSM between the two emotions (positive-negative-emotion RSM). Then, we applied the Fisher r to z transformation to the RSM to ensure a normal distribution of the correlation coefficients. Similarly, we calculated the RSM of the positive (i.e., sympathetic feelings) and negative (i.e., fearful feelings) social emotions of the disease and the control clusters. Then, we applied the Fisher r to z transformation to the RSM of the social emotions. We then calculated the RSM of the personal and social emotions for the high and low empathy groups and tested the discrepancy in pattern similarity between the two groups.

## Results

### Representational Similarity Analysis of Personal Emotions

**FIGURE 1:**
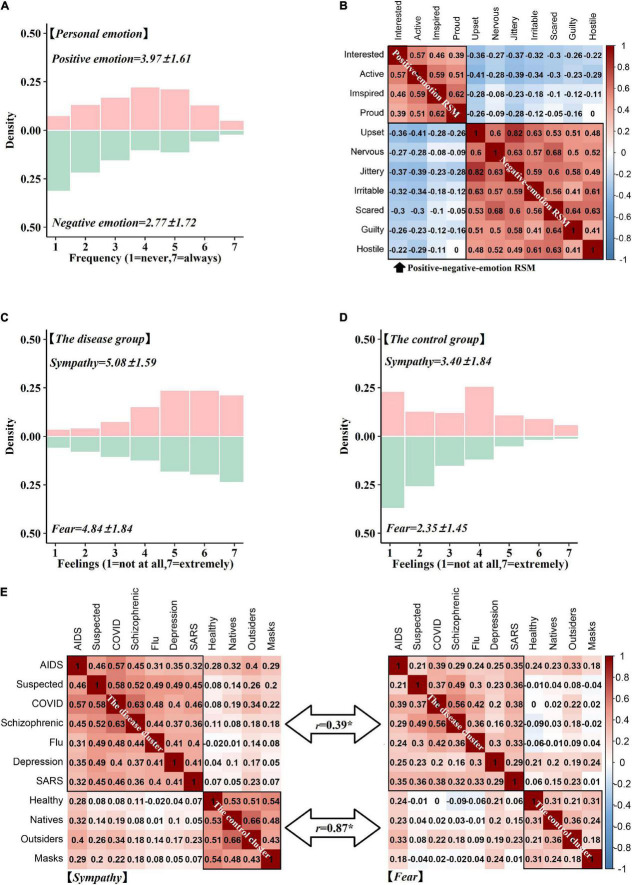
Results of personal and social emotions. **(A)** The histogram of positive (light red) and negative personal emotions (light green); **(B)** The representation similarity matrix (RSM) of personal emotions; **(C)** The histogram of sympathetic (light red) and fearful (light green) feelings for the disease cluster; **(D)** The histogram of sympathetic (light red) and fearful (light green) feelings for the control cluster; **(E)** The RSM of sympathetic and fearful feelings toward the disease and control clusters. *p < 0.5.

We first analyzed the distribution of all emotions (Supplementary Results and Supplementary Figure 2). Generally, participants experienced a low frequency of negative emotions but a medium frequency of positive emotions during the COVID-19 pandemic (Figure 1A). The RSM of the two personal emotions revealed that the positive and negative emotions were inversely correlated with each other in general (Figure 1B).

### Representational Similarity Analysis of Social Emotions

We calculated the distribution of the social emotions of the disease, control, and deviant clusters (Supplementary Results and Supplementary Figure 3). A medium-to-high rate of sympathetic feelings and a similar rate of fearful feelings were observed for the disease cluster (Figure 1C) and lower rates of the two social feelings were shown for the control clusters (Figure 1D). To further examine the pattern similarity of the social feelings, we calculated the sympathy RSM and the fear RSM (Figure 1E) for the two clusters separately. Results showed that the sympathy RSM was correlated with the fear RSM in the disease (*r* = 0.39, *p* < 0.05) and control clusters (*r* = 0.87, *p* < 0.05).

### Personal Representational Similarity Analysis of Individuals With High vs. Low Empathy

**FIGURE 2:**
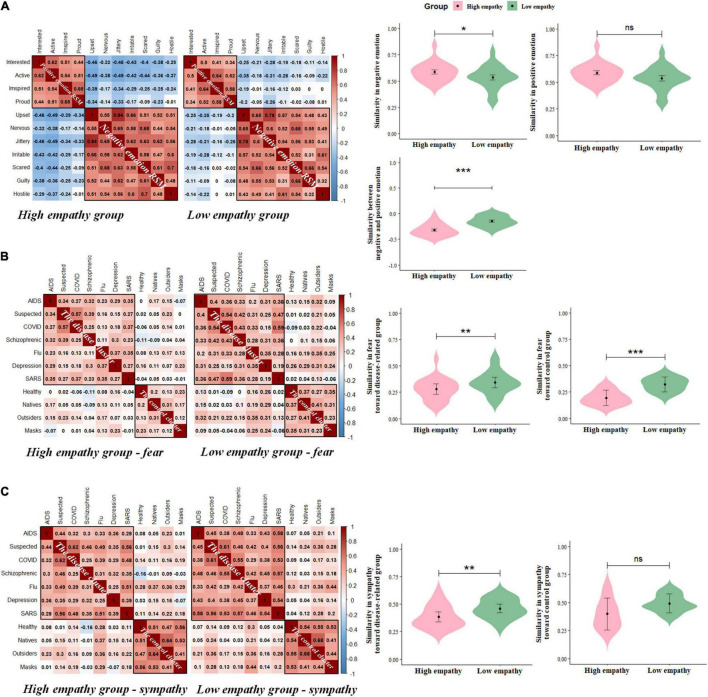
High vs. low empathy in personal and social emotions. **(A)** The representation similarity matrix (RSM) of personal emotions in high and low empathy groups; **(B)** The RSM of fearful feelings toward the disease and control clusters in high and low empathy groups; **(C)** The RSM of sympathetic feelings toward the disease and control clusters in high and low empathy groups. *p < 0.05, **p < 0.01, ***p < 0.001.

First, the distribution results of personal emotions showed that the high (vs. low) empathy group reported a higher frequency of positive and negative emotions (Supplementary Results and Supplementary Figure 4). Consistently, the independent t-tests on each personal emotion item showed that the high (vs. low) empathy group reported a significantly higher frequency of the following emotions: active, upset, nervous, jittery, irritable, scared (2.00 < *ts* < 3.35, *ps* < 0.05, Supplementary Table 1). We then calculated the RSMs of the personal emotions and found more pattern similarity of the negative RSM in the high (vs. low) empathy group [*t*_(20)_ = 3.19, *p* < 0.01, Figure 2A]. No difference in the pattern similarity of the positive RSM between the two groups [*t*_(5)_ = 1.15, *p* = 0.30]. Moreover, the positive-negative RSM of the high empathy group was more dissimilar than that of the low empathy group [*t*_(27)_ = −12.90, *p* < 0.001, Figure 2A], indicating that less pattern similarity between the two personal emotions was induced in the high vs. low empathy group during the pandemic.

### Social Representation Similarity Matrix of Individuals With High vs. Low Empathy

The distribution results showed that the high (vs. low) empathy group showed a higher rate of fear and sympathy for the disease cluster (Supplementary Results and Supplementary Figure 5). Specifically, the independent t-tests showed that the high (vs. low) empathy group reported a higher rate of fear toward COVID-19 patients, AIDS patients, and Flu patients (1.98 < *t* < 2.53, ps < 0.05) and a higher rate of sympathy toward all disease-related people (2.05 < *t* < 3.93, *ps* < 0.05, Supplementary Table 2). Paired sample *t*-test showed that the high (vs. low) empathy group showed less pattern similarity in the fear RSM for the disease [*t*_(20)_ = −2.96, *p* < 0.01] and control clusters [*t*_(5)_ = −12.14, *p* < 0.001, Figure 2B]. In addition, the pattern similarity of the sympathy RSM of the high (vs. low) empathy group was lower for the disease cluster [*t*_(20)_ = −3.14, *p* < 0.01, Figure 2C] but not for the control cluster [*t*_(5)_ = −1.80, *p* = 0.13]. These results suggest that high empathy people may induce more dissimilar and mixed social feelings about the disease-related groups to help them better adjust when facing a pandemic threat.

## Discussion

Previous literature has revealed that individuals’ emotional reactions are related to trait empathy (Powell, 2018; Thompson et al., 2019; Wearne et al., 2019). However, insufficient attention has been paid to the relationship between the trait empathy and different emotions when facing ecological threats. To fill this gap, the current study focused on the pattern similarity of personal and social emotions under the COVID-19 threat and demonstrated that trait empathy strengthens Chinese personal emotions and their social feelings toward a variety of social groups under the pandemic threat. First, people with high (vs. low) trait empathy felt more positive and negative emotions during the recovery stage of COVID-19. The two valences of personal emotions had less similarity in the high (vs. low) empathy group. Second, high (vs. low) empathy people reported more fearful and sympathetic feelings toward the disease-related people. Additionally, the pattern similarity of the two social feelings toward the disease-related groups had less similarity in the high (vs. low) empathy group. To be noted, the conventional analysis (i.e., directly comparing means of emotions) failed to robustly detect the group differences in personal and social emotions (see Supplementary Tables 1, 2). However, the pattern analysis indeed uncovered a robust effect of empathy on modulating Chinese personal and social emotions, which suggests that pattern analysis may be a more powerful tool to capture the pattern of collective emotions under threats.

Previous studies found that there was no association (Spencer-Rodgers et al., 2010) or positive correlation between positive and negative emotions in Asians (i.e., Chinese, Japanese) (Scollon et al., 2005; Cassels et al., 2010). Differently, our results revealed that the two types of personal emotions were negatively correlated, which may be due to the context of the threat. More interestingly, we found that positive and negative emotions were more inversely correlated in high vs. low empathy individuals, suggesting that trait empathy may play a role in enlarging the polarization of the two personal emotions during the pandemic. Moreover, we found that the polarization was driven by the increased pattern similarity of negative emotions in low compared to high empathy individuals. Similarly, Tamborini et al. (1990) found that college students with high empathy traits reacted more strongly to the suffering stimuli when they were watching a horror film. What’s more, high (vs. low) empathy individuals were more sensitive to emotional stimuli (i.e., recognize the emotional state of others from their faces more accurately), especially the negative emotion (e.g., sad and fear) (Chikovani et al., 2015). One possible explanation behind the pattern results of personal emotions is that high empathy relative to low empathy individuals may arouse a higher frequency of a wide range of negative emotions to keep alert when facing ecological threats.

As we have mentioned above, previous studies have found that high empathetic people are more sensitive to threatening stimuli (Fossataro et al., 2016). In line with these studies, our results confirmed that Chinese with high compared to low trait empathy were more likely to fear people with highly infectious diseases and severe mental illness (e.g., COVID-19 patients, schizophrenic patients). The fear of the disease-related people can be explained by the pathogen aversion theory, which holds that aversion to possible pathogen sources serves as a psychological mechanism to protect us from infectious diseases. It is an adaptive ability to avoid potential infection risk by avoiding contact with disease-related groups under the pandemic threat (Park et al., 2003; Oaten et al., 2011). From the evolutionary perspective, empathy helps individuals keep away from things that might harm them. For example, low empathy individuals have more difficulties in inhibiting substance-related addictions (Preller et al., 2013) and behavioral impulsivity (Tomei et al., 2017).

Compared with low empathy individuals, high empathy individuals reported higher levels of empathetic feelings toward the disease group. The reason could be that high empathy individuals could put themselves in others’ situations and understand their emotions, which may facilitate cooperation under threats (Eisenberg and Miller, 1987; Morelli et al., 2014). Previous studies revealed that as a prerequisite for understanding others’ emotional states, high empathy relative to low empathy individuals had a higher ability in detecting and discriminating others’ emotions (Spencer-Rodgers et al., 2010; Olderbak and Wilhelm, 2017). An ERP study found that high empathy individuals induced a larger neural activity in both early (300–600 ms) and late processing (600–800 ms) in discriminating emotional faces (Choi and Watanuki, 2014). Meanwhile, high empathy individuals not only process emotional information deeply but also have different reactions to various targets. For example, high compared to low empathy people had a larger zygomatic activity to angry faces than happy ones (Dimberg and Thunberg, 2012). This finding indicated that high empathy individuals elicited stronger physiological reactions (i.e., muscle activity) in response to others’ negative emotions. Consistent with this, high vs. low empathy people could recognize other people’s emotions more accurately, especially negative emotions (e.g., fear and sadness) (Chikovani et al., 2015). Meanwhile, trait empathy is related to one’s positive feelings (e.g., subjective well-being) (Depow et al., 2021) and promotes prosocial feelings (e.g., willingness to help others) (Telle and Pfister, 2016). Consistent with these studies, our results suggest that Chinese with high empathy traits may experience more mixed social feelings when they think about interacting with disease-related groups under the pandemic threat.

In the current results, people with high empathy have complex social emotions toward disease-related people, that is, sympathy (other-oriented) and fear (self-oriented, based on their avoidance of infection). These results were consistent with the existing research that individuals with high empathy have high self-protection intentions and high other-protection intentions simultaneously at the behavioral level. Specifically, people with high empathy were more willing to take other-protective behaviors during the COVID-19 pandemic (Pfattheicher et al., 2020). Meanwhile, some researchers found that the implementation of pandemic prevention measures in high empathy people was mainly driven by a high self-protection tendency [e.g., Rieger (2020) and Asri et al. (2021)].

The current study has the following limitations. First, the current study revealed that high empathy relative to low empathy people reported more negative and positive emotions during the COVID-19 outbreak (in both personal and social emotions) but showed less pattern similarity in the positive and negative aspects of the two emotions. However, the underlying mechanisms of the group discrepancy in pattern similarity remain unclear. Taking advantage of neuroimaging technology, researchers could further address whether different neural substrates may be recruited to support the modulation of empathy on personal and social emotions. Additionally, the study was conducted during the recovery period of the pandemic. Although the findings have suggested that there are differences in the pattern similarity of personal and social emotions between high empathy and low empathy individuals during the COVID-19 pandemic, we need to take caution in understanding the moderation of empathy in the patterns of emotions in normal times. Future research is encouraged to manipulate different threat levels (vs. non-threat control) to test whether such moderation of empathy is specific to collective threats or not. Another limitation of the current research is that the factors affecting emotions are complex. Therefore, in addition to empathy, whether other factors can affect the pattern of emotions remains unsolved. Considering that individual and social emotions are also linked with other emotion-related traits [e.g., anxiety and neuroticism, Wang et al. (2019); Vinograd et al. (2020), and Brookman et al. (2022)], future work is encouraged to test whether other emotion-related traits or factors would affect the pattern of collective emotions under threats. Moreover, we only focused on two social feelings in this study. It would be interesting to examine the relationship of various social emotions, e.g., guilt, pride, embarrassment, and jealousy.

## Conclusion

The current study highlights the role of empathy trait in modulating the patterns of personal and social emotions during the COVID-19 pandemic. People with high empathy showed more pattern similarity of negative emotions but less pattern similarity of social emotions about the disease-related people than low empathetic people did when facing such a threat. Our findings enrich the existing literature on understanding the role of empathy in mental health and social cognition in the context of threat.

## Data Availability Statement

De-identified data and code of the present study are available upon request to the corresponding author.

## Ethics Statement

The studies involving human participants were reviewed and approved by the Ethics Committees of the Institute of Psychology, Chinese Academy of Sciences. The participants provided their written informed consent to participate in this study.

## Author Contributions

YM conceived of the project. YM and JZ designed the project. JZ implemented the experiments and collected the data. YH and JZ performed the analyses. YH, JZ, and YM wrote the manuscript. All authors discussed the results and approved the submitted version.

## Conflict of Interest

The authors declare that the research was conducted in the absence of any commercial or financial relationships that could be construed as a potential conflict of interest.

## Publisher’s Note

All claims expressed in this article are solely those of the authors and do not necessarily represent those of their affiliated organizations, or those of the publisher, the editors and the reviewers. Any product that may be evaluated in this article, or claim that may be made by its manufacturer, is not guaranteed or endorsed by the publisher.

## Footnotes

1
https://www.wenjuan.com
