# Supplementary material for: Trait Empathy Modulates Patterns of Personal and Social Emotions During the COVID-19 Pandemic
Source: Front Psychol. 2022 Jun 10;13:893328. doi: 10.3389/fpsyg.2022.893328 (PMC9231589; doi:10.3389/fpsyg.2022.893328)
Supplement: Supplementary file 1 [file Data_Sheet_1.pdf]

Supplemental Materials for

Trait empathy modulates patterns of personal and social emotions during the COVID-19 pandemic

This file includes:

Supplementary Results

Table S1-S2

Fig. S1-S6

Supplementary Results

Distribution of personal emotions. The distributions of all negative emotions were right-skewed (0.19 ⩽ skew ⩽ 1.52, *ps* < 0.001, Fig. S2). As Fig 1A illustrated, participants experienced a low frequency of negative emotions in general during the recovery stage of COVID-19 (mean ± SD = 2.77±1.72). Meanwhile, the mean value of the positive emotions was 3.97±1.61. Specifically, inspired (3.63±1.54, skew = 0.15) and proud (3.29±1.65, skew = 0.33) were right-skewed (*ps* < 0.001). However, interested (4.59±1.37, skew = -0.48) and active (4.35±1.52, skew = -0.15) emotions were left-skewed (*ps* < 0.001). In general, Chinese participants reported a relatively low frequency of negative emotions and a high frequency of positive emotions when the pandemic was under control.

Distribution of social emotions. According to the Kolmogorov-Smirnov test, the sympathy and fear distributions of the disease, control, and deviant clusters were skewed (*ps* < 0.001). Specifically, the sympathy distributions of the disease cluster were left-skewed (-1.05 ⩽ skew ⩽ -0.45, *ps* < 0.001, Fig S3A). The mean value of the disease cluster was 5.08±1.59. The sympathy distributions of the control cluster were right-skewed (0.14 ⩽ skew ⩽ 0.65, *ps* < 0.001, Fig S3B) except for native people (skew = -0.06, *p* < 0.001). The mean value of the control cluster was 3.40±1.84 (Fig 1D). The sympathy distributions of the deviant cluster were mainly right-skewed (Fig S3C). The fear distributions of the disease cluster were left-skewed (-1.41 ⩽ skew ⩽ -0.68, *ps* < 0.001, Fig S3A) except for depression patients (skew = 0.25, *p* < 0.001) and for the AIDS patients (skew=0.18, *p* < 0.001). The mean value of the disease cluster was 4.84±1.84. The fear distributions of the control cluster (i.e., healthy people, natives, outsiders, people with masks) were right-skewed (0.40 ⩽ skew ⩽ 1.95, *ps* < 0.001, Fig S3B). In addition, people were less likely to fear the control group (mean ± SD = 2.35±1.45). The fear distributions of the deviant cluster were shown in Fig S3C). Taken together, the distribution results indicated that Chinese participants were likely to generate positive and negative social feelings about people with diseases simultaneously (especially for people with highly infectious diseases and severe mental illness) during the pandemic.

Distribution of personal emotions of individuals with high vs. low empathy. To test the group difference in the distribution of personal emotions, we used the Mann-Whitney *U* test. The mean values of positive/negative emotions were 4.07±1.62/2.98±1.79 for the high empathy group and 3.86±1.59/2.57±1.62 for the low empathy group (Fig S4A). The Mann-Whitney *U* test showed that the high vs. low empathy group reported more positive emotion (inspired: Z = -2.01, *p* = 0.04, Fig S4B) and more negative emotions (upset: Z = -2.28, *p* = 0.02; nervous: Z = -2.43, *p* = 0.02; jittery: Z = -2.49, *p* = 0.01; irritable: Z = -3.17, *p* < 0.01, Fig S4C). No group differences in other emotions were found (Table S1).

Distribution of social emotions of individuals with high vs. low empathy. To test the difference between high and low empathy groups in the sympathy and fear distributions of different social groups, we used the Mann-Whitney *U* test. The mean values of the empathetic feelings towards the disease cluster for the two groups were 5.26±1.55 (high empathy) and 4.73±1.63 (low empathy), respectively (Fig S5A). For the control cluster, the mean values of the sympathetic feelings were 3.51±1.93 (high empathy) and 3.29±1.73 (low empathy) (Fig S5A). The high vs. low empathy group was more likely to sympathize with the social groups in the disease cluster (*ps* < 0.05, Fig S5B). However, they were less likely to feel sympathetic feelings towards social deviants, such as people

not wearing masks (Z = -2.04) and robbers (Z = -2.57) (*ps* < 0.05, Fig S5D). Group differences failed to show for the control cluster (*p* > 0.05, Fig S5C).

The mean values of the fearful feelings towards the disease cluster for the two groups were 5.01±1.82 (high empathy) and 4.75±1.80 (low empathy) (Fig S5A). For the control cluster, the mean values of the fearful feelings of the two groups were 2.24±1.45 (high empathy) and 2.46±1.45 (low empathy) (Fig S5A). Regarding to the disease cluster, the high vs. low empathy group was more likely to fear people related to certain diseases (*ps* < 0.05, Fig S5B), including suspected COVID-19 patients (Z = -2.05), AIDS patients (Z = -2.33), COVID-19 patients (Z = -2.18), and schizophrenic patients (Z = -2.63). The high vs. low empathy group was less likely to fear healthy people (Z = -2.74) and natives (Z = -2.54) (*ps* < 0.01, Fig S5C). As for the deviant cluster, the high empathy compared to the low empathy group was more likely to fear people with no mask (Z = -3.51) and robbers (Z = -3.38) (*ps* < 0.01, Fig S5D).

Supplementary Tables

Table S1. The means and standard deviations of personal positive and negative emotions.

Classification	High empathy(N=171)	Low empathy(N=174)	t	Effect size Cohen's d
Positive emotions				
Interested	4.61±1.46	4.57±1.29	0.27	0.03
Active	4.45±1.57	4.25±1.47	1.24	0.13
Inspired	3.80±1.50	3.47±1.56	2.00*	0.22
Proud	3.41±1.67	3.17±1.63	1.34	0.14
Negative emotions				
Upset	3.82±1.86	3.37±1.70	2.32*	0.25
Nervous	3.21±1.71	2.77±1.57	2.50*	0.27
Jittery	3.47±1.80	3.00±1.69	2.52*	0.27
Irritable	3.07±1.80	2.47±1.55	3.35***	0.36
Scared	2.59±1.67	2.22±1.47	2.17*	0.23
Guilty	2.60±1.69	2.32±1.55	1.64	0.18
Hostile	2.06±1.38	1.86±1.31	1.44	0.16

Note. $^{*}p < 0.05$, $^{***}p < 0.001$

Table S2. The means and standard deviations of social emotions towards the control/disease/deviant cluster.

	High empathy(N=171)		Low empathy(N=174)					
Classification	Fear	Sympathy	Fear	Sympathy	t (fear)	Effect size (fear)	t (sympathy)	Effect size (sympathy)
The Control cluster								
Healthy	1.48±0.90	2.82±1.86	1.78±1.14	2.76±1.74	-2.68**	-0.29	0.28	0.03
Natives	2.11±1.19	3.79±1.86	2.36±1.12	3.67±1.71	-2.01*	-0.22	0.64	0.07
Outsiders	3.10±1.56	3.74±1.79	3.19±1.57	3.41±1.60	-0.55	-0.06	1.80	0.19
People with masks	2.29±1.56	3.69±2.05	2.52±1.63	3.30±1.77	-1.34	-0.14	1.87	0.20
The disease cluster								

AIDS patients	5.30±1.76	4.87±1.74	4.89±1.79	4.48±1.72	2.13*	0.23	2.05*	0.22
Suspected COVID-19 patients	5.60±1.49	5.35±1.36	5.29±1.58	4.80±1.44	1.87	0.20	3.63***	0.39
COVID-19 patients	6.01±1.33	5.75±1.39	5.72±1.47	5.12±1.57	1.94*	0.21	3.93***	0.42
Schizophrenic patients	5.43±1.49	5.19±1.52	5.02±1.56	4.68±1.64	2.53*	0.27	2.96**	0.32
Flu patients	4.18±1.66	4.47±1.58	3.83±1.67	3.96±1.64	1.98*	0.21	2.93**	0.32
Depression patients	3.30±1.70	5.73±1.41	3.40±1.62	5.20±1.52	-0.52	-0.06	3.39***	0.37
SARS patients	5.24±1.77	5.46±1.42	5.10±1.72	4.88±1.58	0.73	0.08	3.61***	0.39
The deviant cluster								
Muslims	3.37±1.80	3.33±1.72	3.18±1.69	2.78±1.66	1.01	0.11	3.00**	0.32
Recovered COVID-19 people	3.74±1.87	5.11±1.58	3.78±1.60	4.61±1.68	-0.21	-0.02	2.82**	0.30
People with no mask	5.22±1.63	2.05±1.41	4.69±1.63	2.36±1.53	3.09**	0.33	-1.92	-0.21
Robbers	6.25±1.24	1.62±1.26	5.72±1.57	1.89±1.36	3.46***	0.37	-1.92	-0.21

Note. $^{*}p < 0.05$, $^{**}p < 0.01$, $^{***}p < 0.001$

Supplementary Figures

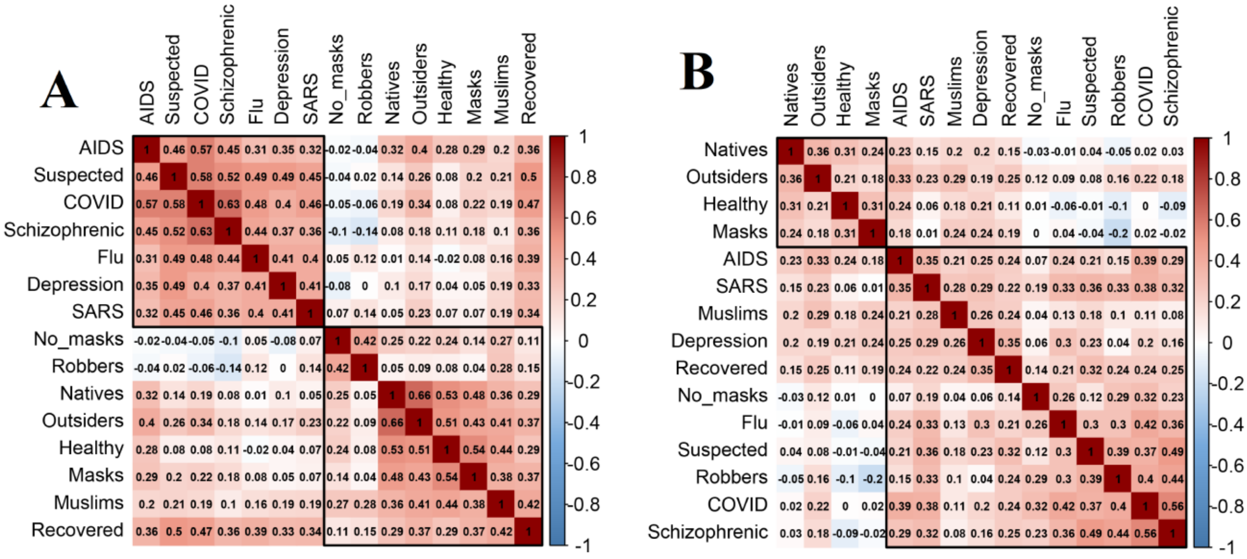

Fig S1. Hierarchical cluster analysis on a set (N=15) of social groups. A) Cluster analysis of the sympathetic feelings; B) Cluster analysis of the fearful feelings.

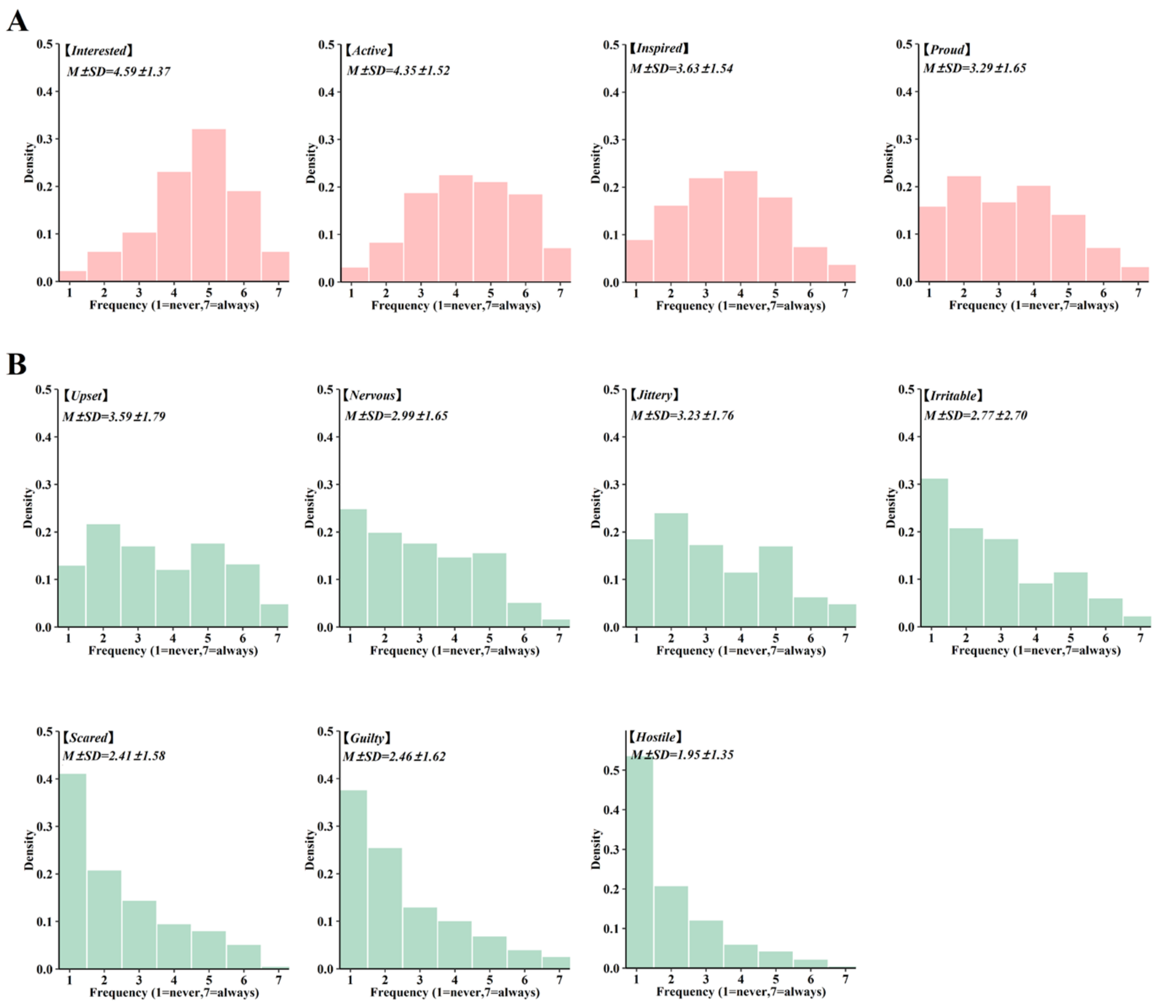

Fig S2. Histograms of personal emotions. A) Positive emotions (light red histograms); B) Negative emotions (light green histograms).

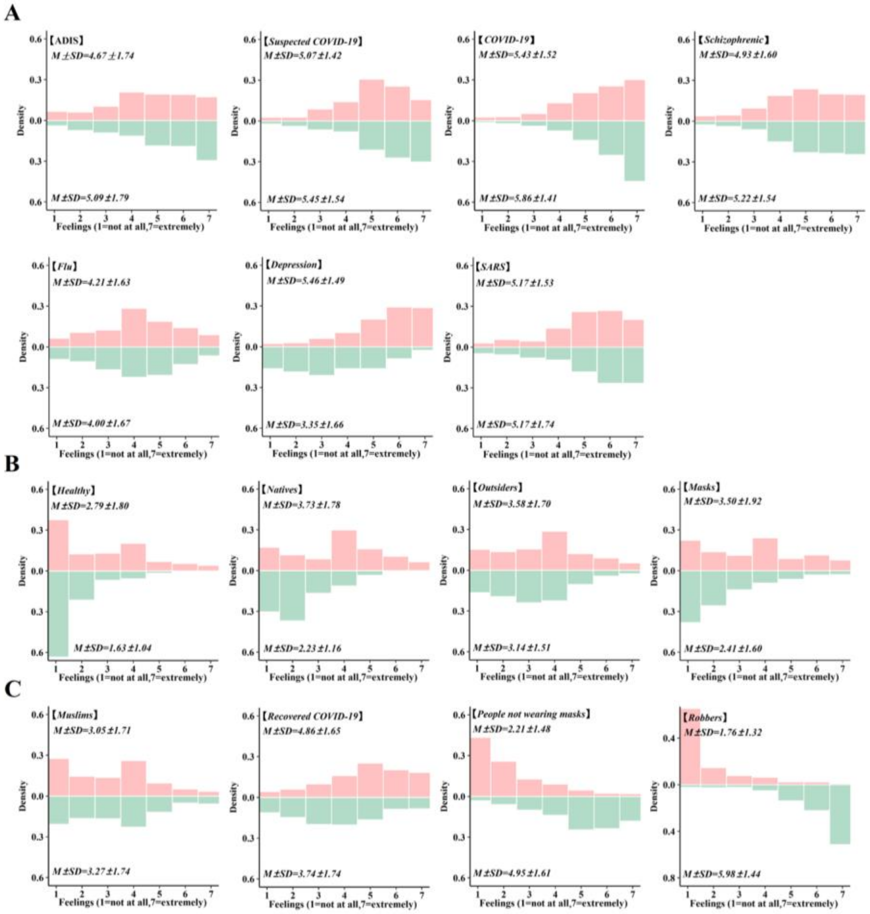

Fig S3. Histograms of social emotions. A) Social emotions toward the disease cluster; B) Social emotions toward the control cluster; C) Social emotions toward the deviant cluster. Red and green histograms represent the sympathetic and the fearful feelings respectively.

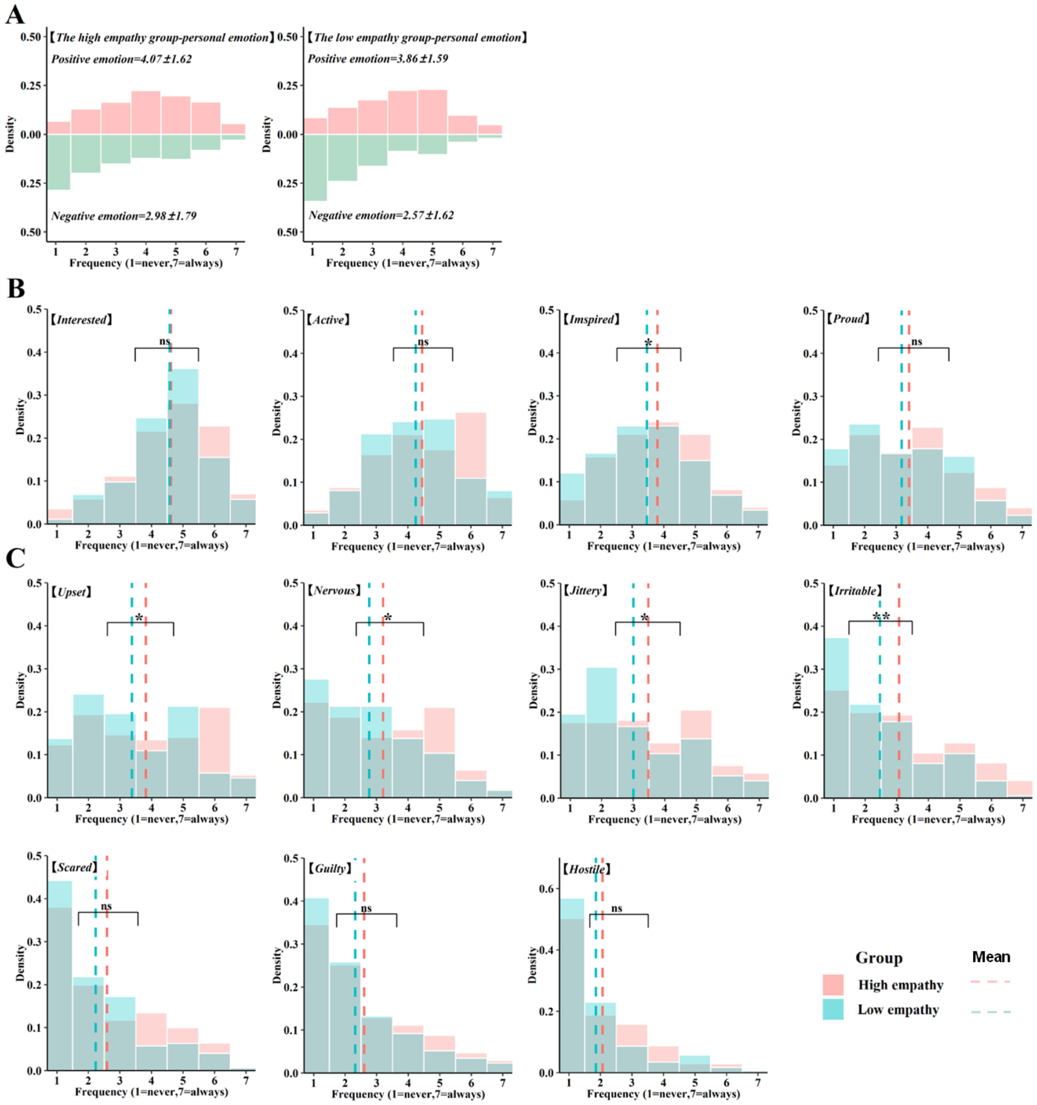

Fig S4. Group differences (high vs. low empathy) in personal emotions. A) The mean histogram of positive and negative emotions of the high and low empathy groups; B) The histogram of positive emotions of the high vs. low empathy groups; C) The histogram of negative emotions of the high vs. low empathy groups. *p < 0.05,**p < 0.01, *ns* >0.5.

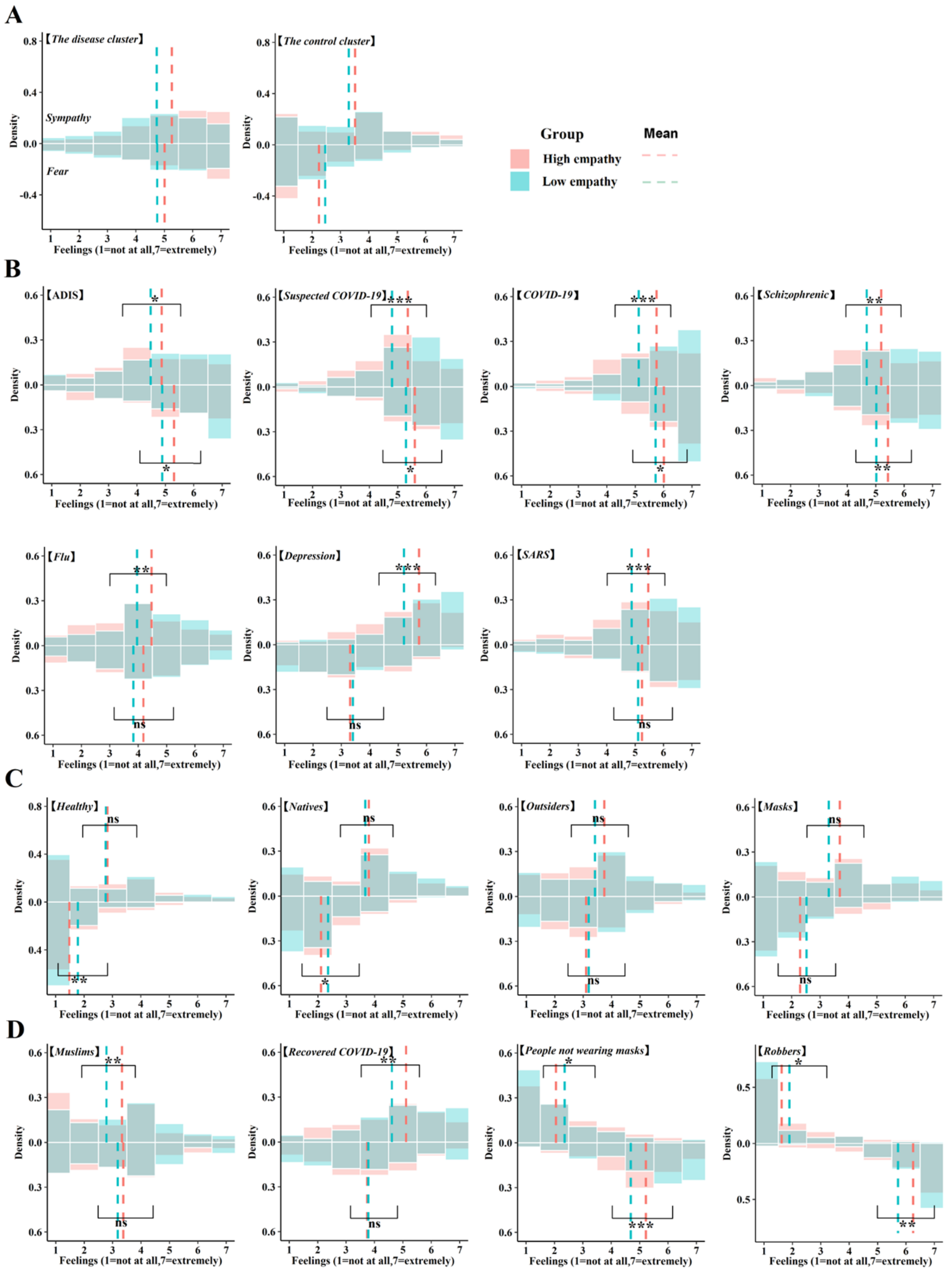

Fig S5. Group differences (high vs. low empathy) in social emotions. A) The mean histogram of fearful and sympathetic feelings towards the disease and control clusters of the high and low empathy groups; B) Social emotions towards the disease cluster of the high vs. low empathy groups; C) Social emotions towards the control cluster of the high vs. low empathy groups; D) Social emotions towards the deviant cluster of the high vs. low empathy groups. * $p < 0.05$,** $p < 0.01$,*** $p < 0.001$, *ns* > 0.5.

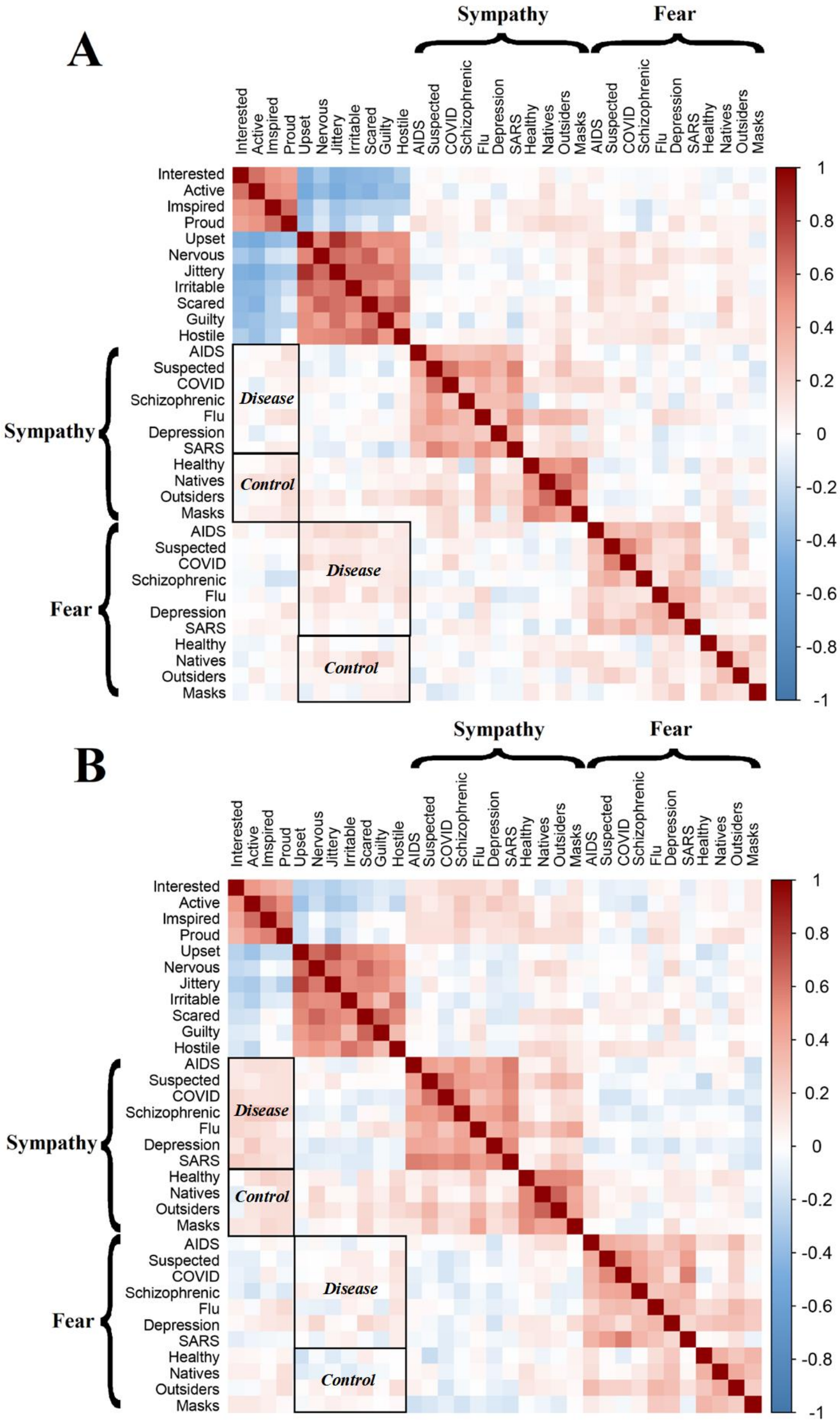

Fig S6. RSM of personal and social emotions in the high (A) and low (B) empathy groups.
